# The Effects of Vitamin D Supplementation on Pulmonary Function of Chronic Obstructive Pulmonary Disease Patients, before and after Clinical Trial

**DOI:** 10.3390/diseases3040253

**Published:** 2015-10-16

**Authors:** Seyed Ali Javad Moosavi, Maryam Haddadzadeh Shoushtari

**Affiliations:** 1 Department of Pulmonary, Iran University of Medical Sciences; Tehran 14174, Iran; E-Mail: Dr_Moosavi_pul@yahoo.ca; 2 Department of Pulmonary, Ahvaz Jundishapur University of Medical Sciences, Ahvaz 61357, Iran

**Keywords:** chronic obstructive pulmonary disease, vitamin D

## Abstract

Vitamin D has several extra calcemic effects. Vitamin D deficiency is highly prevalent in chronic obstructive pulmonary disease (COPD) patients but little is known about it’s association with lung function. Objective: To investigate whether supplementation with vitamin D could improve pulmonary function in COPD patients. Design: Before and after, double center, clinical trial. Setting: Hazrat Rasoul University Hospital, Tehran, and Imam Khomaini University Hospital, Ahvaz, Iran. Participants: 24 patients with mild to very severe COPD. Intervention: Loading dose of 300,000–600,000 International Units (IU) of vitamin D, then 50000 IU weekly for 12 weeks. Measurements: The outcomes included forced expiratory volume in one second (FEV1), forced vital capacity (FVC), vital capacity (VC), forced expiratory flow between 25%–75% of forced vital capacity (FEF 25%–75%), exercise capacity according to the six minute walk test(6MWT) and the saturation of oxygen during exercise. Results: The mean FEV1 (*p*-value = 0.866), FVC (*p*-value = 0.475) and VC (*p*-value = 0.425) were not significantly different before and after intervention. FEF 25%–75% did not improve with this intervention (*p*-value = 0.555). The vitamin D supplementation did not have any significant effect on the exercise capacity (*p*-value=0.175) or the saturation of oxygen (*p*-value = 0.635). Conclusion: Pulmonary function and exercise capacity did not improve with vitamin D supplementation in COPD patients.

## 1. Introduction

Chronic obstructive pulmonary disease (COPD) is characterized by chronic airflow limitation due to inhalation of noxious particles or gases such as cigarette smoke. Airway inflammation is prominent in COPD [1]. With disease progression, FEV1 declines [2]. No treatment has been proven to inhibit COPD progression except smoking cessation [3].

Vitamin D and parathyroid hormone are the regulators of calcium and phosphate but low concentration of vitamin D is associated with many diseases other than osteoporosis, including hypertension, ischemic heart diseases, type I diabetes, cancer, autoimmune diseases and infections. Also, there are significant associations between vitamin D status and death caused by diseases of the respiratory system, the digestive system, and endocrine, nutritional and metabolic diseases [4,5,6,7,8,9,10,11,12,13].

There are some associations between vitamin D deficiency and COPD. Skaaby *et al.* reported an inverse association between vitamin D deficiency and COPD and vitamin D deficiency is present in 60% to 75% of patients with severe COPD [14,15].

There is a strong relationship between serum concentration of 25-hydroxyvitamin D and FEV1 and FVC [16], but to the best of our knowledge there are few studies that have evaluated the effect of vitamin D supplementation on the improvement of lung function in COPD patients.

The aim of this study is to investigate the role of vitamin D supplementation in improving lung function, exercise capacity and the saturation of oxygen during exercise in COPD patients.

## 2. Method and Materials

Our study was a before and after clinical trial. Eligible patients were current or ex-smokers or were exposed to noxious inhalents, and had a diagnosis of COPD according to the Global Initiative for chronic obstructive Lung Disease (GOLD) definition. They had no history of renal stone or renal failure or other diseases that interact with vitamin D metabolism and did not take vitamin D or calcium supplements. They had no recent history of exacerbation before inclusion.

Baseline characteristics included serum levels of 25-hydroxy vitamin D, severity of airflow obstruction, six-minute walk test, and O2 saturation. We excluded patients who needed continued oxygen therapy during exercise, because we did not have suitable portable oxygen for continues oxygen therapy during 6MWT.The spirometery was performed before and after intervention with a standard spirometer (Ganshorn Company, Niederlauer, Germany).

If the patients had a low level of serum 25-hydroxyvitamin D, they took a loading dose of 300,000 of vitamin D for insufficient (<30 ng/mL) and 600,000 IU for deficient (<20 ng/mL) levels intramuscularly and all patients took 50,000 IU weekly for 12 weeks per oral [17,18]. The serum level of vitamin D was checked afterwards and all of the variables were tested again. We evaluated hyper calcuria only in toxic levels of vitamin D (more than 80 ng/mL) [19]. The patients were excluded from the study if they did not want to continue the treatment or were noncompliant.

## 3. Statistical Analysis

We calculated the sample size with consideration of α = 0.05 and β = 0.8. A total of 18 patients were needed for rich statistically significant results. The included patients were 24. One patient died before termination of trial because of severe multi lobar pneumonia. Twenty-three patients continued the study. Paired sample t-test was used to analyze the results.

The patients provided written, informed consent. The study was approved by the local ethics committee of the Iran University of Medical Sciences and registered with Iranian Clinical Trails Center (NCT 2015012720828N1).

## 4. Results

A total of 59 participants aged 35 to 78 years participated in this study. Forty-two patients had insufficient or deficient levels of 25-hydroxyvitamin D. We were able to follow 23 of them to initiate supplementation. The mean age of patients was 62 (SD = 12). Three patients were not cigarette smokers: one was a female water pipe smoker and the two others were exposed to noxious inhalants.

One of the patients was in mild stage, eight in moderate stage,13 in severe and one in very severe stage of COPD. The mean serum level of vitamin D was 14 ng/mL (SD = 5) (Table 1).

**Table 1:** Baseline characteristics.

Characteristic	Value
Men, *n(%)*	20 (83)
Mean age (SD), *y*	64 (11)
**GOLD stage, *n(%)***	
I	1
II	8
III	13
IV	1
Mean 25(OH) vit D	13 (4)
level (SD), ng/mL	

**Figure 1:**
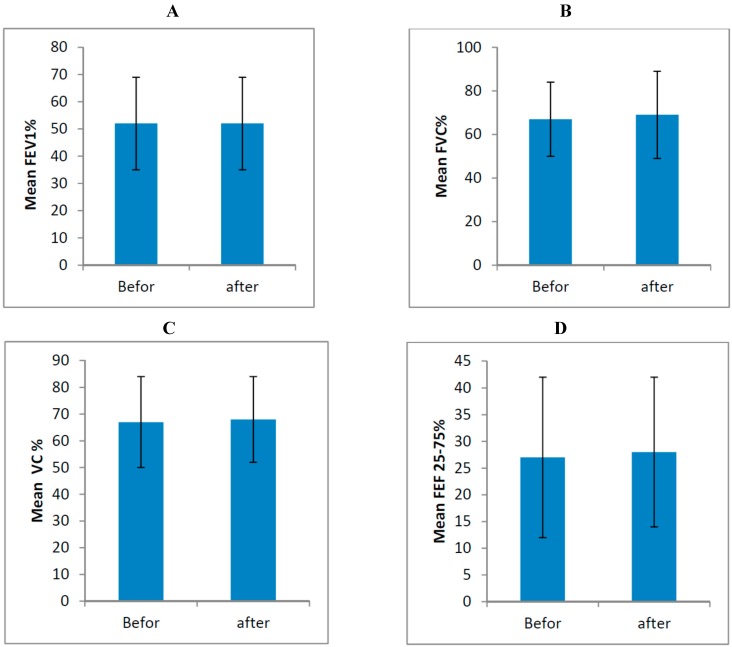
Means of FEV1 (**A**), FVC (**B**), VC (**C**) and FEF 25%–75% (**D**) before and after supplementation.

Paired sample t-test analysis showed no significant differences before and after supplementation between FEV1 (*p*-value = 0.866), FVC (*p*-value=0.475), VC (*p*-value = 0.425) and FEF 25%–75% (*p*-value = 0.555; Table 2) (Figure 1).

The mean distance that participants walked during 6MWT was 350 meters before and 360 meters after vitamin D supplementation, which was not significantly different (*p*-value = 0.175). The saturation of oxygen during 6MWT did not improve with supplementation.

**Table 2:** End points.

Variable	Mean ± SD (Before)	Mean ± SD (After)	*p*-Value
FEV1	52% ± 17%	52% ± 17%	0.866
FVC	67% ± 17%	69% ± 20%	0.475
VC	67% ± 17%	68% ± 16%	0.452
FEF 25%–75%	27% ± 15%	28% ± 14%	0.555
6MWT, m	350	360	0.175
O2 Sat	95% ± 1%	95% ± 1%	0.635

## 5. Discussion

The purpose of this study was to evaluate the role of correction of serum vitamin D level on the pulmonary function of COPD patients. We could not show any effect of this intervention.

A cross-sectional study by Peter N. Black and Robert Scragg reported a relationship between serum 25-hydroxyvitamin D and FEV1 and FVC but there was no difference in the FEV1/FVC ratio between the highest and lowest quintiles of serum level of vitamin D. In spite of a greater difference in FEV1 between the highest and lowest quintiles of vitamin D in those with diagnosis of chronic bronchitis or emphysema than for other participants, the interaction between the diagnosis and the serum vitamin D was not significant [16].

This study had comparable results with a randomized clinical trial by A. Lehouck *et al.* [20]. The main goal of their study was the reduction of COPD exacerbation with a high dose of vitamin D. The annual rate of exacerbation in their study was 2.8. Frequent exacerbations may be associated with a more rapid decline of FEV1 [21] and may interfere with the likely effect of vitamin D supplementation on pulmonary function. Also, most of our participants were screened during outpatient visits and did not have exacerbation long before their inclusion and most of them had no history of hospitalization for COPD exacerbation before and during the study. Only one of our patients had exacerbation at the initiation of the study. However, we used a higher dose of vitamin D supplementation. In fact, there are some suggestions that higher doses of vitamin D are required to increase serum25-hydroxyvitamin D levels, which may be needed for extra calcemic effects [22,23].

The result of a cohort study by K.M. Kunisaki *et al.* supports our study [24].Their cases and controls were continued smokers with rapid and slow lung function decline respectively. The baseline serum 25-hydroxy vitamin D levels were not predictive of subsequent lung function decline in a six-year follow up. Moreover, the results of some trials of vitamin D supplementation on its effect in other chronic diseases have been disappointing [25].

There are some hypotheses for these results. There are several mechanisms of action from vitamin D in COPD: (1) calcemic effects; (2) lung tissue remodeling effects; (3) antimicrobial effects; (4) immune modulation effects; and (5) peripheral muscle function effects.

The calcemic effect of vitamin D on the pulmonary function is related to its kyphosis effect, and its reversal needs a long time period in order to allow for lung tissue remodeling. Therefore, the short term follow up period of our study could not evaluate the vitamin D effects on these mechanisms.

The absence of the immune modulatory effect of vitamin D may be due to the obstruction of small and larger airways in COPD, which is associated with the remodeling process, and seems to be irreversible. The presence of airway obstruction in the absence of accumulation of some type of inflammatory cells in stage 3 and 4 of COPD despite a cessation of smoking nine years earlier supports this idea [1].

There were no significant differences in saturation of oxygen during exercise before and after vitamin D supplementation. This may be confounded by the exclusion of hypoxemic patients for the reason we described. Also, the mean distances that the patients could walk after the intervention were not longer, which supports the results of previous similar studies in heart failure patients [21,23].

Our study had limitations. One was small sample size. In addition, this study was a before and after study. In this type of studies, the intervention is confounded by the Hawthrone effect. This effect can lead to an overestimation of the effectiveness of an intervention. However, we could not show any effect of our intervention and it seems that our results were not confounded by this effect.

The technique of doing 6MWT did not fulfill the ATS guidelines. We did not use the Borg scale of dyspnea and the only end point in this test was the distance that the patient walked without severe dyspnea in his or her sense. Only one of our patients could not continue the test and stopped because of dyspnea.

The other limitation was the short follow up period. Some of our patients were from other cities, or were from villages and we had difficulties with maintaining the study follow up. We believe the likely mechanisms of effect of vitamin D on the outcomes which we described would probably not have prevented us from arriving at our end points in the short follow up period.

In conclusion, a high dose of vitamin D supplementation had no effects on the improvement of pulmonary function and the exercise capacity of COPD patients.
